# Analysis of Erythrocyte C4d to Complement Receptor 1 Ratio: Use in Distinguishing between Infection and Flare-Up in Febrile Patients with Systemic Lupus Erythematosus

**DOI:** 10.1155/2015/939783

**Published:** 2015-07-26

**Authors:** Chen-Hung Chen, Shun-Ban Tai, Hsiang-Cheng Chen, Deng-Ho Yang, Ming-Yieh Peng, Yuh-Feng Lin

**Affiliations:** ^1^Graduate Institute of Clinical Medicine, Taipei Medical University, Taipei 11031, Taiwan; ^2^Division of Rheumatology, Immunology and Allergy, Department of Internal Medicine, Taipei Tzu Chi Hospital, Buddhist Tzu Chi Medical Foundation, School of Medicine, Tzu Chi University, Hualien 970, Taiwan; ^3^Division of Rheumatology, Immunology and Allergy, Department of Internal Medicine, Tri-Service General Hospital, National Defense Medical Center, Taipei 11490, Taiwan; ^4^Division of Rheumatology, Immunology and Allergy, Department of Internal Medicine, Armed-Forces Zuoying General Hospital, Kaohsiung 81342, Taiwan; ^5^Division of Rheumatology, Immunology and Allergy, Department of Internal Medicine, Armed-Forces Taichung General Hospital, Taichung 41168, Taiwan; ^6^Division of Infection, Department of Internal Medicine, Taipei Tzu Chi Hospital, Buddhist Tzu Chi Medical Foundation, School of Medicine, Tzu Chi University, Hualien 970, Taiwan; ^7^Division of Nephrology, Department of Medicine, Shuang Ho Hospital, Taipei Medical University, New Taipei 23561, Taiwan

## Abstract

*Objective.* Fever in systemic lupus erythematosus (SLE) can be caused by infection or flare-up of the disease. This study aimed to determine whether the ratio of the level of erythrocyte-bound C4d to that of complement receptor 1 (C4d/CR1) can serve as a useful biomarker in the differentiation between infection and flare-up in febrile SLE patients. *Methods.* We enrolled febrile SLE patients and determined the ratio on the day of admission. The patients were divided into 2 groups according to the subsequent clinical course. *Results.* Among the febrile SLE patients, those with flare-up had higher ratios and lower C-reactive protein (CRP) levels than those with infection. Cut-off values of <1.2447 and >4.67 for C4d/CR1 ratio and CRP, respectively, were 40.91% sensitive and 100.0% specific for the presence of infection in febrile SLE patients; similarly, cut-off values of >1.2447 and <2.2, respectively, were 80% sensitive and 100% specific for the absence of infection in febrile SLE patients. *Conclusion.* The C4d/CR1 ratio is a simple and quickly determinable biomarker that enables the differentiation between infection and flare-up in febrile SLE patients at initial evaluation. Further, when combined with the CRP level, it is useful to evaluate disease activity in SLE patients with infection.

## 1. Introduction

Systemic lupus erythematosus (SLE) is a common autoimmune disease. Fever in SLE patients can be caused by a number of reasons, with infection and flare-up being the most common. The clinical presentation of SLE flare-up may mimic that of infection coincident with SLE, and the two situations may be difficult to differentiate in febrile patients. Differential diagnosis of fever in SLE is crucial for the optimal management of these patients.

Traditional biomarkers for the survey of disease activity in SLE include anti-dsDNA antibodies and serum complement proteins C3 and C4. However, most SLE patients exhibit persistently high levels of anti-dsDNA antibodies or low levels of complement proteins C3 and C4. Therefore, these biomarkers are insufficient for differentiating disease flares from infection. Several biomarkers can be used to survey susceptibility, establish diagnosis, evaluate disease activity, and assess specific organ involvement in SLE [1, 2]. Among them, the novel biomarkers to evaluate disease activity include serum cytokines, soluble cytokine receptors, soluble cell surface molecules (CD27, CD154, and BAFF) [3], endothelial activation markers (soluble vascular adhesion molecule [sVCAM], soluble intercellular adhesion molecule [sICAM], and thrombomodulin) [4], and cell markers (plasma cell CD27 and erythrocyte-C4d) [5–7]. However, these biomarkers are totally not reliable for practical application to distinguish between active disease and infection.

C-reactive protein (CRP) is a serological parameter conventionally used to distinguish SLE flare-up from infection. Although patients with SLE relapse have an increased erythrocyte sedimentation rate (ESR), their CRP level does not robustly increase, whereas SLE patients with infection exhibit increase in both ESR and the CRP level. However, the CRP level is not always elevated in SLE patients with infection at initial admission, and it may increase in SLE flare-up patients without infection. Therefore, CRP alone is not a reliable parameter to identify infection in patients with SLE [8]. Other soluble biomarkers that can be used to differentiate infectious disease from exacerbation of SLE include reduced expression of soluble Fc gamma receptor III; elevated levels of granulocyte colony-stimulating factor; and elevated levels of sCD14, sICAM-1, sE-selectin [9, 10], and procalcitonin (PCT) [11]. However, some of these tests are carried out only by some medical centers and turnaround times and accuracy of the results can widely vary. PCT is the precursor of the calcitonin, and it is synthesized in the parafollicular C-cells of the thyroid. Serum PCT level increases in severe bacterial and fungal infections, but it may not increase, or increase only slightly, in viral infections [11, 12]. The presence of elevated levels of PCT raises the suspicion of a concurrent bacterial or mycotic infection in patients with active autoimmune diseases. However, no association has been noted between the activity of SLE and PCT levels [13].

Recently our studies found that reduced levels of erythrocyte CR1 may reflect disease activity in lupus patients by using specific monoclonal antibody CR1-2B11 [14, 15]. From previous study reports, increased erythrocyte-bound C4d (E-C4d) was also a useful marker for lupus disease activity except in condition with haemolytic anemia (HA) and chronic renal failure (CRF) [6, 16]. Theoretically we can combine those two markers as indicator for lupus activity determination. In this study, we aimed to identify useful biomarkers for instantly differentiating between infection and flare-up in febrile SLE patients at initial admission. We sought to examine the clinical applicability of the expressions of complement splitting product C4d and complement receptor 1 (CR1) on erythrocytes as a convenient “real value” for clinical application. Our results indicate that the C4d/CR1 ratio can serve as a predictor of infection in febrile SLE patients, thereby enabling the differentiation between infection and flare-up in febrile SLE patients. Most importantly, in the presence of both infection and disease flare-up in febrile lupus patient, this indicator can help determine the appropriate therapy strategy.

## 2. Materials and Methods

### 2.1. Study Participants

All study participants were ≥18 years and provided written informed consent. None of the patients was excluded from participation on the basis of sex or ethnicity. The participants included febrile SLE patients, febrile patients without SLE, and healthy controls. The criteria for the classification of the patients are provided below. The study protocol was approved by the Tri-Service General Hospital Institutional Review Board.

### 2.2. Fever Definition

Fever, in this study, was defined as an ear temperature of over 37.8°C measured on the first hospital day, by using an electronic thermometer.

### 2.3. SLE Patients

Blood samples were collected on the first day of admission, from 47 febrile SLE patients who met the 1982 ACR revised criteria for the classification of definite SLE. This group of patients included 40 women and 7 men, with ages ranging from 18 to 89 y (mean age: 42.28 ± 3.4 y). Disease activity was evaluated in each of these patients in terms of the SLE disease activity index (SLEDAI) score. The E-C4d and E-CR1 levels were measured by flow cytometry. Serum level of anti-dsDNA was determined by enzyme-linked fluorescent immunoassay (Phadia ImmunoCAP System, Phadia GmbH, Freiburg, Germany). The sera C3, C4, and CRP levels were quantitated by means of immunonephelometry on the Behring nephelometer systems (BN II). The patients also underwent laboratory tests to measure the following parameters: erythrocyte sedimentation rate (ESR); white blood cell count; haemoglobin level; platelet count; and serum levels of blood urea nitrogen, creatinine, anti-dsDNA, anticardiolipin IgG/IgM, anti-Sm, anti-Ro, anti-La, anti-RNP, and rheumatoid factor. SLE flare-up was defined as the elevation of over 3 points at admission from the latest preadmission SLEDAI score, without evidence of infection.

### 2.4. Non-SLE Patients

Twenty febrile patients with diseases other than SLE were recruited. For the reason of E-C4d levels being of limited use in evaluation of disease activity in lupus patients with HA and in lupus patients with CRF, febrile non-SLE patients with HA or CRF were also not enrolled. All of those enrolled patients had fever associated with cellulitis, pulmonary tuberculosis, pneumonia, urinary tract infection, virus infection, and so forth.

### 2.5. Healthy Controls

Thirty healthy individuals were recruited as controls. These participants were required to complete a brief questionnaire regarding previous or current medical conditions.

### 2.6. Flow Cytometric Characterization of Erythrocytes

3 mL blood sample was obtained from each study participant at the time of the study visit. Blood samples were placed in Vacutainer tubes (BD Pharmingen, Franklin Lakes, NJ, USA) containing ethylenediaminetetraacetic acid (EDTA) and then shifted to 3 other study tubes. In the first tube, 5 *μ*L of whole blood was incubated with 50 *μ*L of TS1/22, an IgG1 anti-CD11a antibody; this served as the isotype control. Similarly, in the secondary tube, 5 *μ*L of whole blood was incubated with 50 *μ*L of mouse anti-human C4d monoclonal antibody (Quidel, San Diego, CA, USA; 1 mg/mL) at a dilution of 1 : 200. Again, 5 *μ*L of whole blood was added to the third tube and incubated with 50 *μ*L of anti-CR1 monoclonal antibody 2B11 (5 *μ*g/mL) at a dilution of 1 : 250. CR1-2B11 was a kind gift from Dr. L. B. Klickstein (Boston, MA) [14]. After incubation for 30 min, the cells were washed twice with 1 mL of diluent buffer and centrifuged at 1500 ×g for 3 min at 4°C. Before flow cytometric characterization of erythrocytes, 1 *μ*L of fluorescein isothiocyanate- (FITC-) conjugated goat anti-mouse immunoglobulin-specific polyclonal antibody (BD Pharmingen; 500 *μ*g/mL) was added to the supernatant for 30 min at 4°C. The cells were then washed twice again, as described previously, and resuspended in 1 mL of phosphate-buffered saline (PBS). Finally, the samples were analysed by flow cytometry by using a FACSCalibur flow cytometer (Becton Dickinson Immunocytometry System, San Jose, CA, USA). Erythrocytes were electronically gated for 30,000 cells on the basis of their forward- and side-scatter properties. Surface expression of E-CR1 and E-C4d on the gated erythrocytes was reported as specific mean fluorescence intensity (sMFI). The ratio of C4d expression to CR1 expression (C4d/CR1) was calculated as follows: (sMFI of C4d − sMFI of isotype control)/(sMFI of CR1 − sMFI of isotype control).

After determining the C4d/CR1 ratio for all patients on the first day of admission, the patients were divided into 3 groups according to their subsequent clinical course and laboratory test results: SLE with infection (*n* = 22), SLE with flare-up without infection (*n* = 25), and non-SLE with fever (*n* = 20).

### 2.7. Statistical Analysis

SPSS version 15.0 (SPSS, Inc., Chicago, IL) was used for the statistical analyses. Differences in the median values of the participating groups were compared by using the nonparametric Mann-Whitney test. Spearman's rank correlation was applied to detect correlations among the study parameters. A *P* value less than 0.05 was considered statistically significant. Receiver operating characteristic (ROC) curve, a graphical plot of the sensitivity (true positive rate) and specificity (true negative rate) versus false positive rate (1 − specificity or 1 − true negative rate) and false negative rate, was used in our study to determine the cut-off points for CRP and C4d/CR1 ratio that afforded maximum sensitivity and specificity to distinguish between febrile SLE patients with infection and those without infection.

## 3. Results

The clinical and laboratory manifestations of febrile SLE patients with and without infection are shown in Table 1. Fourteen SLE patients with infection had flare-up at initial evaluation. However, their C4d/CR1 ratios were not elevated, which indicated the absence of disease flare-up (Table 2, Patients 1, 2, 3, 7, 9, 11, 13, 16, 18, and 20). Among the 8 patients without increased SLEDAI scores, elevated C4d/CR1 ratio (>0.8731) was noted in 2 patients (Table 2, Patients 5 and 6), which indicated that these 2 patients had disease flare-up concurrent with infection.

**Figure 1:**
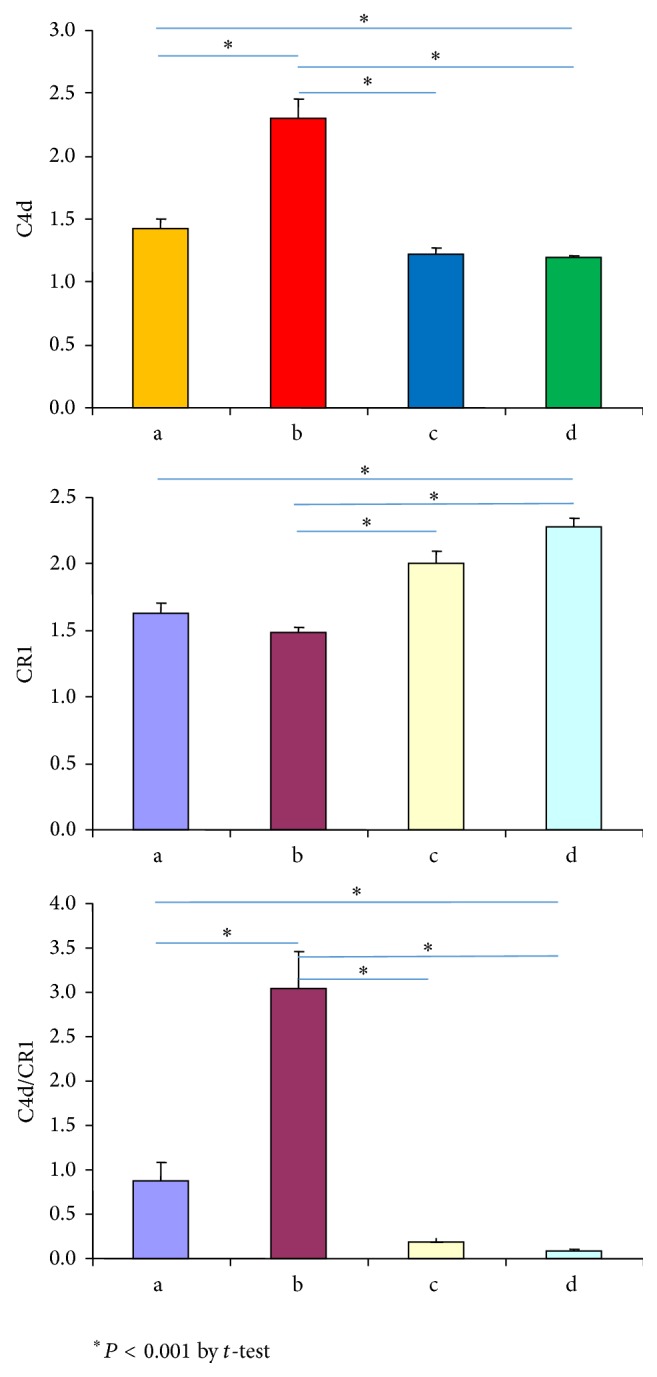
Comparison of the levels of E-C4d and E-CR1 expression and the C4d/CR1 ratio among groups a, b, c, and d. a: febrile SLE patients with infection, b: febrile SLE patients without infection, c: non-SLE febrile patients with infection, and d: healthy controls.

**Figure 2:**
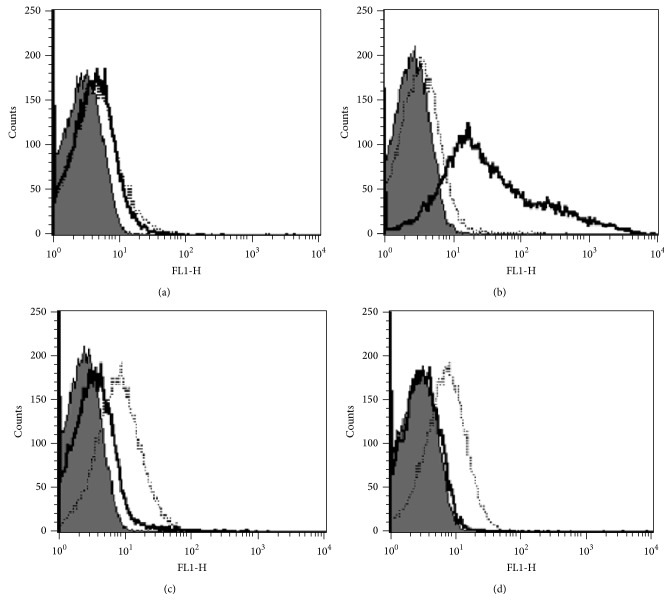
Flow cytometric analysis of E-C4d and E-CR1 expression in (a) febrile SLE patients with infection, (b) febrile SLE patients without infection, (c) non-SLE febrile patients with infection, and (d) healthy controls. Erythrocytes were stained with anti-C4d (black lines, open histogram), CR1-2B11 (dashed lines, open histogram), and isotype-matched control antibodies (solid gray histogram).

Febrile SLE patients without infection had a higher C4d/CR1 ratio than those with infection (3.34 ± 2.17 versus 0.80 ± 0.91, *P* < 0.001). The range of the C4d/CR1 ratio in the febrile SLE patients without infection was 0.68–8.80 and that in the febrile SLE patients with infection was 0.03–3.51 (Table 3, Figure 1). Among the SLE patients, 25 (20 women and 5 men; mean age: 35.44 ± 9.24 y) did not have infection and did not receive any antibiotic therapy, while 22 (20 women and 2 men, mean age: 50.05 ± 16.88 years) did show evidence of viral or bacterial infection and received the therapy (Table 3). Representative flow cytometry staining for each group is shown in Figure 2.

SLE patients with flare-up had significantly higher serum anti-dsDNA and E-C4d levels and lower CRP levels than those with infection (Table 3). However, among the patients with SLE, the E-CR1 expression level slightly differs between those with infection and those with flare-up (*P* = 0.037). The C4d/CR1 ratio was the highest in febrile SLE patients without infection (*P* < 0.001). Further, the C4d/CR1 ratio was significantly different between febrile SLE patients with infection and febrile non-SLE patients and between the former and healthy controls (*P* < 0.001) (Figure 1).

**Figure 3:**
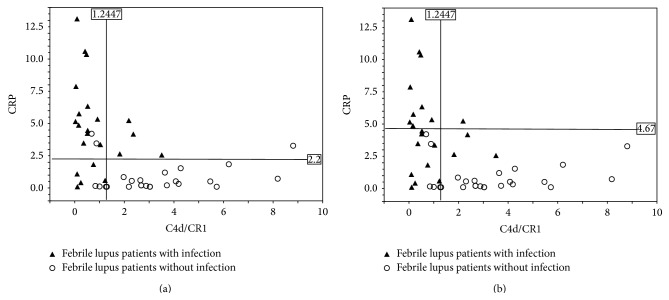
Detection of C4d to CR1 ratio and CRP on erythrocytes (E) from febrile SLE patients with infection and febrile SLE patients without infection. Cut-off points determined by receiver operating characteristic (ROC) curve are indicated by solid lines.

We used receiver operating characteristic (ROC) curve to assess the utility of the assessed parameters in differentiating between febrile SLE patients with infection and those without infection. Sensitivity of 40.91% and specificity of 100.0% were recorded for the presence of infection in febrile SLE patients when the cut-off values of <1.2447 and >4.67 were applied to the C4d/CR1 ratio and serum CRP level, respectively (Table 4, Figure 3); similarly, sensitivity of 80% and specificity of 100% were noted for cut-off values of >1.2447 and <2.2, respectively, for the absence of infection in febrile SLE patients (Table 5, Figure 3).

## 4. Discussion

C4d, a degradation product of C4, can bind with various cells, including reticulocytes and platelets, in the peripheral circulation, but they bind mostly with erythrocytes. Patients with SLE show increased expression of erythrocyte-bound C4d, which serves as a diagnostic tool and indicator of disease activity in SLE [6, 16–18]. CR1 (CD35)—a membrane receptor for C3b and C4b expressed on erythrocytes, leukocytes, and podocytes [14, 15, 19]—plays an important role in the removal of immune complexes and pathogens coated with C3b and C4b [20]. An abnormally low erythrocyte CR1 level is considered characteristic of SLE [15]. Although erythrocyte-bound C4d is a useful biomarker to predict and monitor SLE disease activity, the detected levels of C4d expression vary across laboratories because of the differences in the fluorescence-conjugated antibodies used; this reduces the utility of this marker in clinical settings. In this study, we combined those two markers which indicated the concomitant C4d deposition and CR1 consumption on erythrocyte to obtain a “ratio,” and we sought to evaluate the usefulness of this ratio as a single indicator for differentiating between infection and flare-up in febrile SLE patients. The usefulness of this ratio is not influenced by the variation in the fluorescence-conjugated antibodies used by different laboratories.

Fever is usually caused by exogenic pyrogens; most often, they are infected by bacteria and their endotoxins, viruses, yeasts, spirochetes, and protozoa. Infection is a common problem and has become one of leading causes of mortality in SLE patients and fever is a common manifestation of SLE infection or flare-up. Therefore, differential diagnosis of several SLE flare-up syndromes from infection-related conditions is important [21]. We noted a significant difference of the C4d/CR1 ratios between groups: febrile SLE patients without infection had significantly higher C4d/CR1 ratios than those with infection at initial admission (*P* < 0.001, Table 3). Therefore, the C4d/CR1 ratio can serve as a useful marker to differentiate between fever caused by infection and that caused by flare-up in SLE patients.

The pathogenesis of SLE involves a whole range of factors, including genetic and environmental factors [22]. Infections may play a pivotal role in the expression of the disease in genetically susceptible individuals and can serve as environmental triggers that induce or promote the development of SLE in such individuals [23]. In SLE patients, infection may trigger disease flare-up, and, sometimes, disease flare-up may be confused with infection. A broad spectrum of infections has been reported in SLE patients; these include bacterial, mycobacterial, viral, fungal, and parasitic infections, with the respiratory and urinary tracts being the most commonly involved sites [24]. Among the infections, urinary tract infection (UTI) has been reported as the most common primary or secondary infection in SLE patients, followed by respiratory tract infection.* Escherichia coli* is the most frequent organism identified in culture studies of the tissue samples of SLE patients. The clinical manifestations of UTI are variable, ranging from asymptomatic UTI to urosepsis [25]. In our study, 22 patients with SLE had infection, including urinary tract infection (UTI, *n* = 8), respiratory tract infection (*n* = 5), cutaneous and soft tissue infection (*n* = 2), gastrointestinal tract infection (*n* = 2), peritonitis (*n* = 1), sepsis (*n* = 1), and viral infection (*n* = 3).

The causative pathogens identified in our SLE patients included the following:* Escherichia coli*,* Lactobacillus* spp.,* Candida* spp.,* Pseudomonas aeruginosa*,* Streptococcus pneumoniae*,* Mycoplasma* spp.,* Klebsiella pneumoniae*,* Shigella sonnei*,* Enterococcus faecalis*,* Staphylococcus aureus*, cytomegalovirus (CMV), and herpes simplex virus. In our study, the most frequently isolated pathogens were* Escherichia coli* and* Candida*.* Salmonella* spp. were also common pathogens identified. SLE patients with* Salmonella* infection are at high risk of mortality [26]. CMV infection has been associated with the exacerbation of SLE [27], and the mechanisms by which CMV may trigger autoimmunity have been proposed [28]. Of the 2 patients in our study who were infected by CMV, one had high C4d/CR1 ratio; the possible reason for this may be cooccurrence of CMV infection and SLE flare-up, which suggests that CMV infection can act as a potential trigger for SLE flare-up.

In clinical settings, it is difficult to ascertain whether fever in SLE patients is caused by infection combined with a flare-up. In this study, we used the SLEDAI as a tool to evaluate disease flare-up. The SLEDAI was developed in Canada and covers 24 items, including 16 clinical characteristics and 8 items based solely on laboratory results (urinary casts, haematuria, proteinuria, pyuria, hypocomplementemia, increased DNA binding, thrombocytopenia, and leukopenia). Unfortunately, this index focuses on new or recurrent manifestations and fails to account for clinically important manifestations of ongoing disease activity, such as haemolytic anaemia [29]. In our study, some febrile SLE patients had definitive evidence of infection and also elevated C4d/CR1 ratio, indicating the presence of flare-up with infection. However, their SLEDAI scores at initial assessment did not indicate SLE flare-up. In contrast, high SLEDAI scores were obtained in patients with low C4d/CR1 ratios in patients with definitive infection. According to the clinical presentation and posttreatment outcomes, the C4d/CR1 ratio appears to be more accurate than the SLEDAI score in evaluating disease activity in febrile SLE patients.

Thus, when SLE patients exhibit elevations of both serum CRP level and C4d/CR1 ratio on admission, the cooccurrence of infection and disease flare-up may be suspected. When serum CRP level increases in SLE patients without elevation of the C4d/CR1 ratio, it is likely that the patients have only infections and not flare-up. On the contrary, when only C4d/CR1 ratio is elevated in febrile SLE patients, the cause of fever is mostly SLE flare-up. We found that cut-off values of <1.2447 and >4.67 for C4d/CR1 and serum CRP level, respectively, were sufficient to distinguish febrile SLE patients with infection (40.91% sensitive and 100.0% specific) (Table 4) from febrile SLE patients without infection. Further, the cut-off values of >1.2447 and <2.2 for C4d/CR1 and serum CRP level, respectively, were 80% sensitive and 100% specific for the absence of infection in febrile SLE patients (Table 5).

There are some limitations to this study. In SLE patients with HA, the C4d/CR1 ratio may be higher than expected, which may lead to overestimation of disease activity; in these patients, the C4d/CR1 ratio is too high to differentiate between flares-up and infections. Oppositely, lower C4d/CR1 may be observed and lead to underestimation in patients with CRF. Thus, this novel biomarker may not be suitable to monitor disease activity in SLE patients with HA or CRF [6]. The mechanism underlying the increased levels of active complement fragment in SLE patients with HA remains unclear.

In conclusion, the C4d/CR1 ratio is a simple and quickly determinable biomarker to differentiate between infection and flare-up in febrile patients with SLE. Further, it is a useful marker for follow-up assessment of febrile SLE patients with infections who manifest disease flare-up later in the clinical course. Furthermore, regular monitoring of this ratio in SLE patients can facilitate the assessment of disease activity and recognize infection, in case it occurs subsequently.

## Figures and Tables

**Table 1:** Comparison of clinical and laboratory manifestations of the febrile SLE patients with and without infection.

Manifestation	Febrile SLE patients with infection (*n* = 22)		Febrile SLE patients without infection (*n* = 25)	
	*n*	%	*n*	%
Convulsion	0	0	0	0
Psychosis	1	5	2	8
Mental organic syndrome	0	0	0	0
Cerebrovascular event	1	5	1	4
Vasculitis	0	0	0	0
Arthritis	2	9	3	12
Myositis	1	5	0	0
Urinary casts	0	0	5	20
Haematuria	8	36	11	44
Proteinuria	6	27	13	52
Pyuria	9	41	12	48
Exanthema	0	0	1	4
Alopecia	0	0	0	0
Oral ulcers	3	14	4	16
Pleuritis	3	14	5	20
Pericarditis	2	9	5	20
Complement decrease	10	46	22	88
DNA increase	6	27	13	52
Thrombocytopenia	3	14	6	24
Leukopenia	1	5	7	28

**Table 2:** Clinical pathogens and characteristics of patients with SLE and infection.

	Infectious disease	Pathogen	CRP	SLEDAI before admission	SLEDAI on admission	Proposed SLE flares (SLEDAI increased by ≧3)	C4d/CR1 ratio
1	UTI	*Escherichia coli *	10.4	1	5	Yes	0.4761
2		*E. coli *	13.1	2	8	Yes	0.0974
3		*E. coli *	5.16	1	8	Yes	0.0341
4		*Lactobacillus *	0.59	1	14	Yes	1.2286
5		*Candida *	2.56	4	5	No	3.5118
6		*Candida *	5.34	1	3	No	0.92
7		*Candida *	6.34	1	6	Yes	0.5254
8		*Klebsiella pneumoniae *	4.24	4	6	No	0.5143

9	Pneumonia	*Pseudomonas aeruginosa *	4.87	4	10	Yes	0.1613
10		*Streptococcus pneumoniae *	2.65	1	24	Yes	1.8182
11		*Mycoplasma *	0.1	1	5	Yes	0.1111
12		*Mycoplasma *	1.67	2	3	No	0.1
13		H1N1	10.6	6	14	Yes	0.4182

14	Peritonitis	*Klebsiella pneumoniae *	3.37	3	17	Yes	1.0384

15	Infectious diarrhoea	*Shigella sonnei *	7.88	1	1	No	0.054
16		*Shigella sonnei *	3.49	1	5	Yes	0.36

17	Sepsis	*Enterococcus faecalis *	5.76	1	2	No	0.175

18	Cellulitis	*Staphylococcus aureus *	5.24	1	14	Yes	2.1818
19		*Staphylococcus aureus *	0.42	1	1	No	0.25

20	Viral infection	CMV	4.47	2	5	Yes	0.5238
21		CMV	4.18	1	10	Yes	2.3636
22		Herpes simplex virus	1.83	1	2	No	0.7586

**Table 3:** Clinical characteristics of SLE patients with and without infection.

Variable	Infection (*n* = 22)	Noninfection (*n* = 25)	*P* value^*^
	Mean ± standard deviation		
Male (*n*, %)	2, 9.10%	5, 20.0%	0.423^**^
Age (y)	50.05 ± 16.88	35.44 ± 9.24	0.001
SLEDAI	7.41 ± 6.02	10.48 ± 5.67	0.079
C4d	1.40 ± 0.29	2.30 ± 0.66	<0.001
CR1	1.63 ± 0.31	1.48 ± 0.15	0.037
C4d/CR1	0.80 ± 0.91	3.34 ± 2.17	<0.001
CRP	4.71 ± 3.40	0.85 ± 1.16	<0.001
Anti-dsDNA	116.46 ± 155.21	182.77 ± 173.74	0.177
C3	81.98 ± 31.98	56.86 ± 29.40	0.007
C4	18.29 ± 9.93	13.00 ± 6.15	0.031

^*^ *P* value by *t*-test, ^**^ *P* value by Fisher's exact test.

**Table 4:** Receiver operating characteristic (ROC) curve analysis of the utility of the C4d/CR1 ratio and serum CRP level in febrile SLE patients with infection.

Rules			Number(s)	Sensitivity (%)	Specificity (%)
A	C4d/CR1 > 1.2447	CRP > 4.67	1	4.55	100.0
		CRP < 4.67	3	13.64	12.5
	C4d/CR1 < 1.2447	CRP > 4.67	9	40.91	100.0
		CRP < 4.67	9	40.91	69.2

	Total		22		

B	C4d/CR1 > 1.2447	CRP > 2.2	4	18.18	80.0
		CRP < 2.2	0	0	0
	C4d/CR1 < 1.2447	CRP > 2.2	13	50.09	86.7
		CRP < 2.2	5	22.73	71.4

	Total		22		

**Table 5:** Receiver operating characteristic (ROC) curve analysis of the utility of the C4d/CR1 ratio in febrile SLE patients without infection.

Rules			Number(s)	Sensitivity (%)	Specificity (%)
A	C4d/CR1 > 1.2447	CRP > 4.67	0	0	0
		CRP < 4.67	21	84	87.5
	C4d/CR1 < 1.2447	CRP > 4.67	0	0	0
		CRP < 4.67	4	16	30.8

	Total		25		

B	C4d/CR1 > 1.2447	CRP > 2.2	1	4	20.0
		CRP < 2.2	20	80	100.0
	C4d/CR1 < 1.2447	CRP > 2.2	2	8	13.3
		CRP < 2.2	2	8	28.6

	Total		25		
